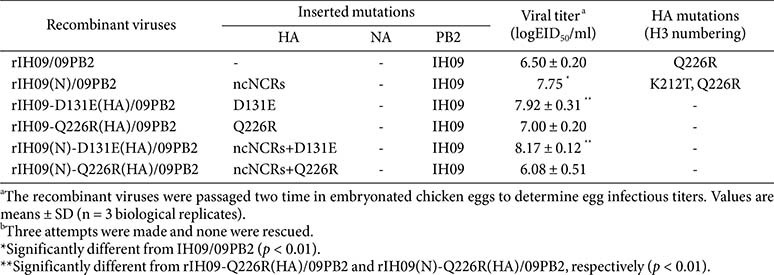# Genetic Optimization of Early 2009 Pandemic H1N1 Vaccine Strains for Improved Replication in Embryonated Chicken Eggs

**DOI:** 10.4014/jmb.2510.10027

**Published:** 2025-12-15

**Authors:** Seung-Eun Son, Jin-Ha Song, Se-Hee An, Chung-Young Lee, Il-Hwan Kim, Ho-Won Kim, Kang-Seuk Choi, Hyuk-Joon Kwon

**Affiliations:** 1 Laboratory of Avian Diseases, College of Veterinary Medicine, Seoul National University, Seoul 08826, Republic of Korea; 2 Avian Influenza Research & Diagnostic Division, Animal and Plant Quarantine Agency, Gimcheon 39660, Republic of Korea; 3 Department of Microbiology, School of Medicine, Kyungpook National University, Daegu 41944, Republic of Korea; 4 Division of Emerging Infectious Diseases, Korea Disease Control and Prevention Agency, Cheongju 28159, Republic of Korea; 5 Research Institute for Veterinary Science, College of Veterinary Medicine, Seoul National University, Seoul 08826, Republic of Korea; 6 Farm Animal Clinical Training and Research Center (FACTRC), Institutes of Green Bio Science and Technology (GBST), Seoul National University, Pyeongchang 25354, Republic of Korea; 7 Laboratory of Poultry Medicine, Department of Farm Animal Medicine, College of Veterinary Medicine and BK21 PLUS for Veterinary Science, Seoul National University, Seoul 08826, Republic of Korea; 8 GeNiner Inc., Seoul 08826, Republic of Korea

**Keywords:** Influenza A virus, pandemic H1N1, untranslated regions, RNA-dependent RNA polymerase, One Health

## Abstract

The 2009 pandemic H1N1 (pdm09) virus is both zoonotic and reverse-zoonotic, transmitting from swine to humans and vice versa. During the early zoonotic phase, immediately after the species jump and before substantial antigenic drift had accumulated, recombinant vaccine strains bearing hemagglutinin (HA) and neuraminidase (NA) from early pdm09 viruses often replicated poorly in embryonated chicken eggs (ECEs), contributing to delays and shortages in vaccine supply. Developing seed strains that are more productive in ECEs while preserving antigenicity and minimizing mammalian pathogenic potential is therefore essential for future pandemic preparedness. Efficient egg replication requires a balanced activity between HA and NA and their coordinated interaction with the polymerase subunit PB2. To this end, we generated PR8-derived recombinants combining PB2 backbones with distinct polymerase activities with targeted HA and NA modifications and edits to segment-specific 3’ and 5’ noncoding regions (NCRs). Comparative analysis of viral titers, together with sequence-based predictions of mutation effects, identified genotypes that improved replication in eggs while minimizing antigenic variations and reducing markers associated with mammalian virulence. Although further enhancement of viral yield is still warranted, these results delineate practical design principles, favoring balanced tuning of HA–NA functions, PB2 compatibility, and NCR context over large receptor-shift mutations, for engineering influenza seed strains. This work provides actionable guidance to support vaccine development and strengthen One-Health-oriented pandemic preparedness.

## Introduction

Influenza A virus (IAV) is an enveloped, negative-sense, single-stranded RNA virus with an eight-segment genome that infects a wide range of avian and mammalian hosts [1]. Genome segmentation, together with an error-prone RNA-dependent RNA polymerase, drives antigenic drift and reassortment, enabling recurrent emergence of novel constellations that breach species barriers [2, 3]. Over the past century, this genetic plasticity has caused repeated epidemics and occasional pandemics with substantial impacts on both human and animal health [4, 5]. The most recent example is the 2009 H1N1 pandemic, in which a novel virus (pdm09) emerged through complex reassortment among avian, swine, and human IAV lineages, ultimately replacing the previously circulating seasonal H1N1 strain [6].

Vaccination remains the cornerstone of influenza control, and egg-based production continues to underpin global supply due to cost-efficiency, scalability, and established infrastructure [7, 8]. Nevertheless, suboptimal replication of particular strains in embryonated chicken eggs (ECEs) can delay vaccine availability, as highlighted in 2009, prompting introduction of egg-adaptive substitutions in hemagglutinin (HA) to enhance binding to avian-type α2,3-linked sialic acids [7, 9]. While such substitutions can raise yields, they may also affect antigenicity and immunogenicity, motivating complementary strategies that preserve vaccine potency [10, 11]. This challenge is directly relevant to veterinary medicine ECEs are central to the introduction of both veterinary and human influenza vaccines.

Reverse genetics enables rational design of seed strains via targeted genomic modifications, ranging from direct HA edits to indirect approaches such as promoter optimization, modification of segment-specific noncoding regions (NCRs), and replacement of internal genes [12-16]. Efficient replication in eggs requires a calibrated balance among polymerase activity, HA-mediated receptor binding, and neuraminidase (NA)-mediated release; perturbing this balance can impair rescue in mammalian cells or reduce yields in ECEs. Each IAV segment bears 3'and 5' NCRs with conserved promoter elements and segment-specific sequences that regulate transcription, replication, and selective genome packaging [17, 18]. In line with this, modifying conserved promoters or segment-specific NCRs has improved the rescue efficiency and replication of PR8-derived recombinants [12-14, 16, 19, 20].

Host adaptation reflects coordinated changes across segments. HA, the principal target of neutralizing antibodies, frequently acquires egg-adaptive substitutions that fine-tune receptor specificity during passage [1, 3, 21]. These changes often co-evolve with NA to maintain the functional balance between virion attachment and release [22]. Beyond the catalytic site, NA’s second sialic-acid binding site (2SBS)—commonly retained in avian viruses but typically lost in mammalian-adapted strains—also modulates this balance, and loss or restoration of 2SBS is frequently offset by compensatory substitutions in HA [23-25]. In parallel, tuning polymerase activity via PB2 exchange, using avian-adapted PB2 or the cognate pdm09 PB2, has been shown to increase egg growth and antigen yield [26, 27].

In our previous study, incorporating cognate pdm09 PB2 enhanced the egg-based yield of a recombinant virus derived from a contemporary pdm09 strain [27]. Whereas contemporary isolates replicated moderately in ECEs, early pdm09 isolates exhibited markedly poorer replication. Since 2009, pdm09-lineage strains have accumulated mutations across HA, NA, and PB2 that reshape their functional interplay. We hypothesized that early pdm09 viruses represent a transitional evolutionary stage in which HA, NA, and PB2 functions were not yet harmonized, necessitating compensatory HA substitutions such as Q226R and D225G for efficient replication of early vaccine candidates [9].

Here, we sought to define the minimal genetic constellation required to generate high-yield, antigenicity-preserving, and safe recombinant vaccine strains from early pdm09 H1N1 viruses. By systematically interrogating HA NCRs, NA 2SBS restoration, HA receptor-binding substitutions, and PB2 compatibility, we aimed to clarify mechanisms underlying poor egg adaptation and to provide practical design rules for next-generation seed strains. These insights advance understanding of pdm09 molecular evolution and support One-Health-oriented preparedness for future influenza emergencies.

## Materials and Methods

### Eggs and Viruses

Specific pathogen-free (SPF) eggs were purchased from VALO Biomedia (Germany), incubated at 37°C for ten days, and then used for all egg-based procedures. 293T cells were acquired from the Korean Collection for Type Cultures (KCTC, Republic of Korea), and maintained in Dulbecco’s Modified Eagle’s Medium (DMEM, Gibco, USA) supplemented with 10% Fetal Bovine Serum (FBS, Gibco) and 1% penicillin-streptomycin (Gibco) at 37°C with 5% CO_2_.

An A(H1N1)pdm09-like clinical isolate representing the early pdm09 reference A/California/04/2009, A/Korea/IH09/2009 (GISAID isolate ID: EPI_ISL_20191996; hereafter IH09) was obtained from diagnostic specimens. Antibiotic-treated samples were diluted in PBS and inoculated into 10-day-old SPF ECEs. After incubation at 37°C for 72 h, allantoic fluid was harvested, and those with positive hemagglutination (HA) titers were aliquoted and stored at −80°C for further analysis.

### Plasmids, Cloning, and Site-Directed Mutagenesis

IH09 HA, NA, and PB2 genes were cloned into the bidirectional reverse-genetics vector pHW2000 as described by Hoffmann *et al*. [28]. Insert sequences were verified by Sanger sequencing using CMV-F and bGH-R primers. Six internal segments (PB2, PB1, PA, NP, M, and NS) of A/Puerto Rico/8/1934 (PR8) virus and the PB2 of A/chicken/Korea/01310/2001 (H9N2) (01310) carrying I66M, I109V, and I133V substitutions (310MVV) previously cloned into pHW2000 vector were used as indicated [29, 30].

Where specified, the 5’ and 3’ noncoding regions (NCRs) flanking IH09 HA (and NA, where indicated) were replaced with the corresponding PR8 NCR sequences by site-directed mutagenesis. Selected egg-adaptive HA substitutions and secondary sialic acid binding site (2SBS)-restoring NA substitutions were introduced into IH09 HA and NA, respectively. Point mutations were generated using a commercial site-directed mutagenesis kit (Muta-Direct Site-Directed Mutagenesis Kit; iNtRON Biotechnology, Republic of Korea) according to the manufacturer’s instructions and confirmed by sequencing with CMV-F and bGH-R primers.

### Generation and Titration of Recombinant Viruses

Recombinant viruses were generated using the eight-plasmid reverse-genetics system in 293T cells as described previously [28]. Briefly, equimolar amounts of the eight pHW2000 plasmids (HA, NA, and six internal genes as specified) were transfected into 293T cells seeded in 6-well plates using Plus Reagent and Lipofectamine 2000 (Life Technologies, USA). At 24 h post-transfection, 1 ml Opti-MEM (Life Technologies) supplemented with 4 μg/well concentration of L-1-tosylamido-2-phenylethyl chloromethyl ketone (TPCK)-treated trypsin (Sigma-Aldrich, USA) was added. A day after, supernatants were harvested and 0.2 ml was inoculated into 10-day-old SPF ECEs, which were then incubated at 37°C for 72 h. Virus rescue was confirmed by hemagglutination (HA) assay using 1%chicken red blood cells (RBCs). The sequences of the mutant viruses were verified by RT-PCR and sequencing.

For growth comparison, allantoic fluids were passaged twice in ECEs unless otherwise stated. Harvested viruses were serially diluted from 10^-1^ to 10^-9^ in 10-fold increments and inoculated into groups of four 10-day-old SPF ECEs per dilution. After incubation for three days at 37°C, the 50% egg infectious dose (EID_50_) was calculated using the Spearman–Karber method [31].

### RT-PCR, Sequencing, and Comparative Sequence Analysis

The full genome sequences of the propagated viruses were confirmed via RT-PCR followed by Illumina MiSeq sequencing (Illumina, USA), which was performed by Bionics Co., Ltd. (Republic of Korea), as described previously.

Viral RNA was extracted from the allantoic fluids using the Patho Gene-spin DNA/RNA Extraction Kit (iNtRON Biotechnology). All eight vRNA segments were amplified by one-step RT‒PCR (SuperScript IV One-Step RT-PCR System; Invitrogen, USA) with universal primers MBTuni-12 and MBTuni-13, and products were assessed by agarose gel electrophoresis [32]. Libraries were prepared and sequenced on an Illumina MiSeq platform (Bionics Co., Ltd., Republic of Korea) followed by analysis using Geneious Prime (v.2023.2.1., Biomatters Ltd.). Reads were trimmed using the BBDuk script, merged, and de novo assembled. The contigs were annotated by BLAST (https://blast.ncbi.nlm.nih.gov/). To identify egg-adaptive substitutions, IH09-derived HA and NA sequences were aligned to the parental IH09 reference sequences using ClustalW multiple alignment tool of the BioEdit program (v7.2.5).

For broader comparisons, we retrieved HA, NA, and PB2 protein sequences from avian H1N1 viruses and pdm09-lineage H1N1 vaccine-reference strains from the GISAID EpiFlu database (accessed 2025-02-14). Only complete sequences passing GISAID quality filters were included. The avian H1N1 dataset comprised n = 711 unique sequences collected from 1975 to 2025. The pdm09 set included WHO vaccine-reference strains (*e.g.*, A/California/07/2009, A/Guangdong-Maonan/SWL1536/2019, and A/A/Victoria/2570/2019). Multiple sequence alignments were generated with Clustal Omega in Geneious Prime (v2023.2.1., Biomatters Ltd.), and consensus sequences and residue frequencies were compared as described.

### Mini-Genome Assay

Polymerase activity was assessed using a Pol I-driven firefly luciferase vRNA-like reporter (pHW-NP-Luc) as described [29]. 293T cells seeded in 12-well cell culture plates were co-transfected with 20 μg/μl of plasmids encoding PB2 (PR8 PB2, 310MVV, or IH09 PB2), PB1, PA, and NP (PR8), together with pHW-NP-Luc and 0.1 μg of the *Renilla* luciferase control plasmid pRL-TK (Promega, USA) to normalize variations in transfection efficiency. At 24 h post-transfection, cells were lysed and luminescence was measured using the Dual-Glo Luciferase Assay System (Promega) on a TECAN Infinite 200 Pro plate reader (Tecan Benelux bv, Netherlands) following the manufacturer's instructions. Firefly activity was normalized to *Renilla* and expressed relative to the polymerase activity of PR8 PB2. Unless otherwise indicated, three independent experiments were performed with technical duplicates.

### Statistical Analysis

Viral titers and polymerase activities were analyzed by one-way ANOVA analysis followed by Tukey’s multiple comparisons test, unless otherwise indicated (GraphPad Prism version 10.0.2 for Windows, GraphPad Software, USA). Fig. 2 data were analyzed by two-way ANOVA followed by Šídák’s multiple comparisons test. The results were considered statistically significant if *P* < 0.05. For each dataset, the exact test used and the number of biological replicates (n) are reported in the corresponding figure legend or table footnotes.

## Results

### Non-Conserved 3’ and 5’ Non-Coding Regions (ncNCRs) of pdm09 HA Genome Hinder Recombinant Virus Rescue

**Fig. 1:**
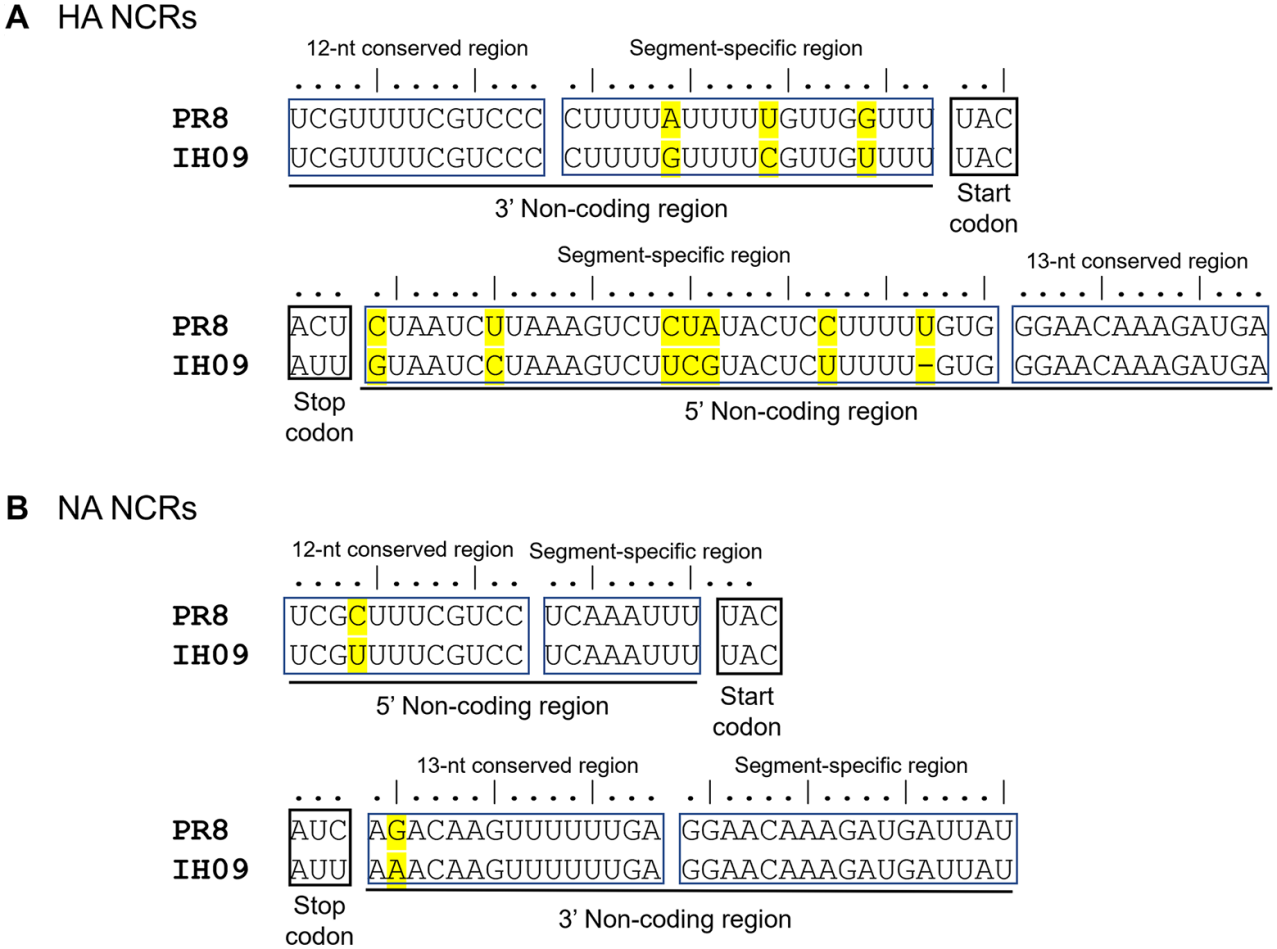
Sequence comparison of 3’ end and 5’ ends non-coding regions of HA gene. IH09; A/Korea/IH09/2009 strain, PR8; A/Puerto Rico/8/1934 strain.

We attempted to generate a PR8-derived recombinant pdm09 H1N1 virus carrying the HA and NA genes of A/Korea/IH09/2009 (IH09). Sequence analysis revealed that the HA of IH09 shared high similarity (up to 99.82%identity) with other 2009 pandemic isolates but contained a rare, strain-specific substitution (T106I, H3 numbering). For virus generation in this study, the HA was reverted to the consensus residue (I106T). Initial reverse-genetics attempts in 293T cells were unsuccessful, as the targeted recombinant virus (rIH09) could not be rescued after three successive passages in embryonated chicken eggs (ECEs). Given our prior observations on the negative impact of non-conserved noncoding regions (ncNCRs) on recombinant virus rescue, we compared the ncNCR sequences of IH09 HA and NA with those of PR8, and identified multiple nucleotide differences in the HA ncNCRs, whereas the NA ncNCRs were nearly identical to those of PR8 (Fig. 1) [16]. Replacing the IH09 HA ncNCRs with those of PR8 enabled successful recovery of the recombinant virus, rIH09(N) (Table 1).

Despite the rescue, rIH09(N) replicated poorly in ECEs (10^4.25^ EID_50_/ml) and acquired the D131E substitution in HA during the passages. These findings suggest that modification of HA ncNCRs can facilitate rescue of recombinant viruses from 293T cells that otherwise fail to replicate in ECEs, potentially by permitting or accommodating compensatory HA mutation.

### Combination of ssNCRs Mutation and Avian 2SBS Restoration Induce Q226R Mutation in HA

Unlike most human and swine viruses, avian H1N1 neuraminidases (NAs) retain a functional second sialic acid-binding site (2SBS). Sequence comparison of the 2SBS regions in avian and pdm09 H1N1 viruses showed that the NA of pdm09 viruses maintained two of the three key contact residues, but had a serine-to-asparagine substitution (S369N) at the third contact residue in the 370-loop (Table 2). To assess the effect of fully restoring 2SBS activity on viral replication in ECEs, we replaced the altered residues in the 370 and 400 loops of IH09 NA with the corresponding avian viral sequences. This modification markedly increased the viral titer of rIH09(N)-2SBS(NA) to 10^7.75^EID_50_/ml, which is approximately 1,000-fold higher than rIH09(N), and was consistently accompanied by the acquisition of the Q226R mutation in HA (Table 1).

To determine the contribution of Q226R to viral replication, we generated recombinant viruses carrying Q226R alone or in combination with the 2SBS modification and compared their titers (Table 1). The titer of rIH09(N)-Q226R(HA)+2SBS(NA) (10^7.83^EID_50_/ml), which carries both the Q226R and 2SBS mutations, was 10-fold higher than that of rIH09(N)-Q226R(HA) (10^6.83^EID_50_/ml) carrying only the Q226R mutation, and similar to that of rIH09(N)-2SBS(NA), which shares the same genetic background. Thus, avian 2SBS synergistically increased virus titer together with ncNCRs and Q226R mutations. These results indicate that restoration of the avian 2SBS, in the presence of ncNCRs modification, is associated with the emergence of the receptor-binding–enhancing Q226R mutation in HA, potentially to counterbalance increased NA activity and thereby improve replication in ECEs.

### Q226R Mutation Alone Is Sufficient for Virus Rescue and Enhanced Replication

To evaluate the contribution of individual HA mutations to virus rescue and replication, we introduced either D131E or Q226R into PR8-derived IH09 recombinants (Table 1). Introduction of D131E mutation alone yielded rIH09-D131E(HA), which replicated at titers comparable to rIH09(N) (10^4.00^ EID_50_/ml). However, when combined with the ncNCRs modification, rIH09(N)-D131E(HA) exhibited approximately 10-fold higher titer (10^5.25^ EID_50_/ml), indicating an additive effect between D131E and ncNCRs changes.

By contrast, introduction of the Q226R mutation alone enabled efficient rescue and produced rIH09-Q226R(HA), with markedly higher titers (10^7.50^ EID_50_/ml) than those of rIH09(N). Notably, combining rIH09 with ncNCRs modification did not further enhance replication, as rIH09(N)-Q226R(HA) exhibited titers similar to rIH09-Q226R(HA). Taken together, these results suggest that while D131E and ncNCRs are synergistic to improve replication, the Q226R mutation alone is sufficient to promote robust virus rescue and high titers in ECEs.

### The ncNCRs of IH09 HA Is Also Incompatible with the Avian-Origin PB2 (310MVV)

To assess the effect of avian-origin PB2 on the replication of mutated recombinant IH09 strains, we replaced PR8 PB2 with PB2 derived from avian H9N2 virus (A/chicken/Korea/01310/2001; 310MVV) and generated a panel of recombinant viruses (Table 3). Interestingly, rIH09/310MVV was not rescued, and 01310MVV PB2 may be also incompatible with ncNCRs of IH09 HA. The viral titer of rIH09(N)/310MVV (10^7.25^ EID_50_/ml), which acquired the D131E substitution in HA, was approximately 1,000-fold higher than that of rIH09(N) carrying the same mutation, and comparable to that of rIH09(N)/310MVV with the D131E substitution introduced intentionally (10^7.5^ EID_50_/ml).

In contrast, combining ncNCRs and 2SBS modifications in the 310MVV background introduced an additional HA substitution (S165I) in rIH09(N)-2SBS(NA)/310MVV, but the resulting titer (10^5.67^ EID_50_/ml) was significantly lower than those of other 310MVV-bearing mutant strains. Furthermore, neither ncNCRs and Q226R combination [rIH09(N)-Q226R(HA) /310MVV] nor ncNCRs, Q226R and 2SBS combinations [rIH09(N)-Q226R(HA)+2SBS(NA)/ 310MVV] showed further increases in titers (10^7.08^ EID_50_/ml) compared to their counterparts with PR8 PB2. However, combination of ncNCRs and 2SBS with Q226R mutation [rIH09(N)-Q226R(HA)+2SBS(NA)/310MVV] displayed a significantly higher titer than rIH09(N)-2SBS(NA)/310MVV carrying S165I.

These findings suggest that incorporation of 310MVV PB2 can substantially alter replication outcomes depending on the accompanying HA and NA modifications, and that differences in PB2 polymerase activity (Fig. S1) may underlie the variable titres observed among strains with otherwise similar genetic backgrounds.

### Cognate IH09 PB2 with HA D131E Is the Minimal Requirement for a Replicative, Antigenicity-Preserving, Safe Vaccine Strain

**Fig. 2:**
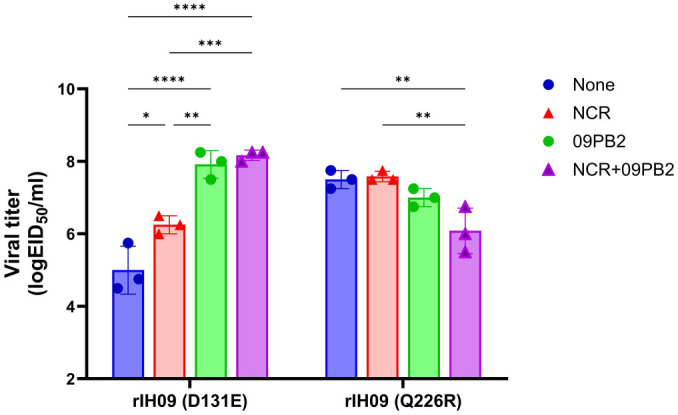
The effect of D131E and Q226R mutations and NCR+PB2 modification on viral titers. Viral titers were measured for recombinant viruses differing in HA residue 226 (D131E vs. Q226R) and NCR+PB2 status (None, NCR only, 09PB2 only, NCR+09PB2). Data represent means ± SD of three independent biological replicates per group. Statistical significance was analyzed by two-way ANOVA followed by Šídák’s multiple comparisons test (*p < 0.05, **p < 0.01, ***p < 0.001).

Polymerase activity assays demonstrated that IH09 PB2 (abbreviated as 09PB2) had significantly lower activity than PR8 PB2, but a level comparable to that of 310MVV (Fig. S1). To evaluate the effect of the cognate PB2 on replication, we replaced PR8 PB2 with IH09 PB2 in the recombinant IH09 strains (Table 4 and Fig. 2). Unlike rIH09, the virus rIH09/09PB2 was successfully rescued without ncNCRs modification and acquired the Q226R mutation in HA (Table 4). Incorporation of ncNCRs modification (rIH09(N)/09PB2) significantly increased the viral titer by more than 10-fold (10^7.75^ EID_50_/ml) and induced additional HA mutation, K212T, together with Q226R.

To directly compare the effects of HA substitutions, we generated rIH09-D131E(HA)/09PB2 and rIH09-Q226R(HA)/09PB2 (Table 4 and Fig. 2). Although not significant, the titer of rIH09-D131E(HA)/09PB2 (10^7.92^ EID_50_/ml) was approximately 10-fold higher than that of rIH09-Q226R(HA)/09PB2 (10^7.00^EID_50_/ml), although the latter still exceeded that of the genetically similar rIH09/09PB2 (10^6.50^ EID_50_/ml) carrying only Q226R. Addition of ncNCRs modification to D131E [rIH09(N)-D131E(HA)/09PB2] resulted in the highest titers (10^8.17^ EID_50_/ml) with significant difference compared to other viruses, but not significantly different from rIH09-D131E(HA)/09PB2 (Fig. 2). In contrast, addition of ncNCRs modifications to Q226R mutation [rIH09(N)-Q226R(HA)/09PB2] resulted in lower titer (10^6.08^ EID_50_/ml) relative to rIH09-Q226R(HA)/09PB2 (Fig. 2).

These results indicate that the D131E and ncNCR modifications are compatible and contribute additively to virus replication, whereas Q226R and ncNCRs mutations are incompatible. This incompatibility was also observed in the corresponding PR8-derived IH09 strains (Table 1).

## Discussion

To date, many PR8-derived recombinant viruses have failed to be rescued, but the underlying reasons remain unclear. Our findings suggest that incompatibility between PR8 PB2 (or 310MVVPB2) and the non-conserved non-coding regions (ncNCRs) of IH09 HA hampers virus rescue. This incompatibility can be partially compensated by HA mutations that reduce binding affinity for mammalian receptors in 293T cells. A plausible explanation is that mismatched ncNCRs impair segment incorporation or RNA synthesis, delaying viral replication after cell entry [13, 17, 20]. Although native IH09 HA likely supports rapid attachment and entry into mammalian 293T cells due to higher affinity for α2,6-linked receptors, subsequent progeny production appears delayed, consistent with a potential mismatch between PR8-derived PB2 and IH09 HA ncNCRs. In contrast, introducing avian receptor–preferring substitutions into IH09 HA is expected to slow entry into 293T cells, increasing the residence time of extracellular virions in culture; however, the same changes may favor replication in embryonated chicken eggs (ECEs), where α2,3-linked receptors predominate, thereby improving overall yields. This interpretation aligns with prior reports showing that replacing segment ncNCRs with the corresponding PR8 ncNCRs enhanced virus titres of PR8-derived H5N1 and H7N9 vaccine strains in ECEs, presumably by optimizing packaging signals and transcription/replication efficiency under egg-based conditions [13, 14].

Nevertheless, the accumulation of mammalian-pathogenic mutations in PR8 PB2 underscores the need for safer PB2 backbone [27, 33, 34]. Although rIH09(N)-2SBS(NA), rIH09(N)-Q226R(HA)+2SBS(NA) and rIH09-Q226R(HA) carrying PR8 PB2 showed improved virus titres, they remain unsuitable as vaccine seed strains. Prior studies also indicate that reassortment between pdm09 and seasonal H1N1 strains is restricted by incompatibility of polymerase subunits, particularly PB2 and PB1 [35]. Our results further suggest that incompatibility between seasonal PB2 genes and pdm09 HA ncNCRs may serve as an additional barrier, thereby limiting the emergence of reassortants carrying pdm09 HA and NA together with more virulent seasonal PB2 genes [36]. Although vaccine production is conducted under strict biosecurity, the development of strains that balance replication efficiency with safety remains a critical priority [33, 34]. In this regard, introducing PR8-derived ncNCRs could inadvertently bypass the natural incompatibility barrier between seasonal PB2 and pdm09 HA, making such an approach less preferable. Instead, constructs such as rIH09-D131E(HA)/09PB2 may represent a safer and more effective alternative compared with PR8-based reassortants like rIH09(N)-D131E(HA)/09PB2.

We previously showed that replacing PR8 PB2 with a cognate PB2 from recent pdm09 strain is sufficient to generate high-yield recombinant vaccine viruses in ECEs [27]. By contrast, early pdm09 isolate tested here was less satisfactory, consistent with observations during the initial 2009 outbreak [9]. These early strains appear to have been in a transitional state, where HA, NA, and polymerase functions were not yet balanced, thereby favoring the emergence of compensatory HA mutations such as Q226R or K212T/Q226R. Notably, these mutations are absent from avian progenitors but frequently selected during egg passage [37]. In contrast, more recent pdm09 strains have accumulated stabilizing mutations in HA and NA that enhance both α2,3- and α2,6-binding, reduce NA activity, and promote antigenic drift, resulting in a more balanced interaction among the viral segments (Table S1) [38-40]. As a result, recombinant viruses bearing HA and NA from these recent pdm09 strains replicated efficiently in embryonated chicken eggs (ECEs) after simple replacement of PR8 PB2 with a strain-matched cognate PB2, without requiring major additional mutations (Table S1) [27].

Comparisons with avian H9N2 further highlight the role of HA-NA balance in egg adaptation. Following their introduction into poultry, Korean-endemic Y439-lineage H9N2 strains initially replicated poorly in ECEs and required multiple serial passages before they could be used as vaccine strain. During this process, successive loss of the NA stalk (18 amino acids) and acquisition of compensatory HA mutations occurred, likely to fine-tune the functional interplay between HA and NA [41]. In the case of IH09, restoration of the NA second sialic acid–binding site (2SBS), particularly through the N369S substitution in the 370-loop, enhanced NA binding to avian receptors and coincided with selection of Q226R in HA (Table 1).

In contrast, pdm09 NA appears to have evolved in the opposite direction. Recent substitutions at the 2SBS contact residues (N369K and K432E) were shown to decrease binding affinity to avian receptors and impair virus release from polyvalent sialoproteins (fetuin) containing both avian- and mammalian-type receptors [42, 43]. Moreover, although pdm09 viruses lack NA stalk deletions, early strains retained an N-linked glycosylation motif (NQS, residues 50–52) that was subsequently lost in later strains such as GD19 and Vic/21 (Table 2). While we did not experimentally confirm its effect here, loss of N-glycans adjacent to the stalk are generally associated with decreased NA activity [44, 45]. Thus, the combined effect of glycosylation loss and 2SBS substitutions may have reduced NA activity in recent pdm09 strains, thereby shifting the balance with HA and PB2. Whether the introduction of specific substitutions such as N50D and/or K432E into early pdm09 NA could optimize this balance and improve replication in eggs remains an open question.

Egg-adaptive HA mutations displayed distinct patterns depending on the presence of ncNCRs, 2SBS, and different PB2 backbones. Among them, Q226R is a well-characterized mutation that shifts receptor specificity toward avian receptors by replacing an uncharged glutamine with a positively charged arginine [37]. While this enhances binding to α2,3-linked receptors, it concurrently reduces α2,6 binding and alters antigenicity. By contrast, D131E increases receptor binding avidity to both human- and avian-type receptors by introducing spatial flexibility without major structural changes, and has been linked to enhanced replication and increased virulence in murine models [46]. Notably, whereas Q226R is predominantly reported in egg-adapted pdm09 viruses, D131E is widely found among H1N1 avian influenza viruses as well as early seasonal H1N1 lineages, highlighting its broader role in viral fitness [47-49]. Our results suggest that although Q226R confers a strong entry advantage, it may impose a replication bottleneck when not accompanied by sufficient polymerase activity. In contrast, D131E supports more balanced replication, as evidenced by consistently high viral titers of D131E-carrying viruses regardless of NCR status. Importantly, Q226R has also been associated with antigenic variation that can compromise vaccine efficacy [11]. Located outside the major antigenic site Sa, prior work indicates that D131E produces minor antigenic changes while modulating receptor binding in pdm09 backgrounds [49]. While direct antigenic, immunogenicity, and protection readouts lie beyond the scope of this study, the available evidence suggests that conservative substitutions such as D131E are less likely than Q226R to disadvantage immunogenicity or protection. Furthermore, enhanced avian-type receptor binding has been reported to induce nonspecific interactions with glycoproteins such as immunoglobulin Y in allantoic fluid, leading to epitope masking during inactivation [25]. In this context, the rIH09-D131E(HA)/09PB2 combination represents a promising intermediate vaccine strain-combining improved replication with preserved antigenicity and lower biosafety risk compared with Q226R/PR8 PB2-based strains.

In conclusion, our study demonstrates that the replicative fitness of pdm09-lineage H1N1 viruses in eggs is determined by the interplay between HA, NA, and PB2. Early pdm09 isolates were in a translational state, necessitating compensatory HA and NA mutations for efficient rescue and replication, whereas recent strains have accumulated mutations that provide relatively improved balance among viral segments. Nonetheless, egg-adaptive mutations remain frequent, particularly in HA, underscoring the ongoing challenge of producing vaccine strains without undesirable antigenic changes. Among the mutations tested, D131E offered a favorable profile by enhancing replication particularly in combination with cognate PB2. While these findings highlight balanced genetic modifications as a rational strategy for developing high-yield, antigenically stable, and safe egg-based influenza vaccine strains, the present study is limited by its focus on a subset of genetic backgrounds and by the absence of direct antigenic and immunogenicity assessments. Future work should therefore evaluate the stability of these candidate strains across diverse genetic constellations, assess their antigenic integrity using serological and immunization studies, and investigate whether fine-tuning NA activity in combination with D131E and cognate PB2 can further enhance replicative fitness without compromising vaccine efficacy.

## Supplemental Materials

Supplementary data for this paper are available on-line only at http://jmb.or.kr.



## Figures and Tables

**Table 1:** Characterization of PR8-derived mutant recombinant IH09 strains.

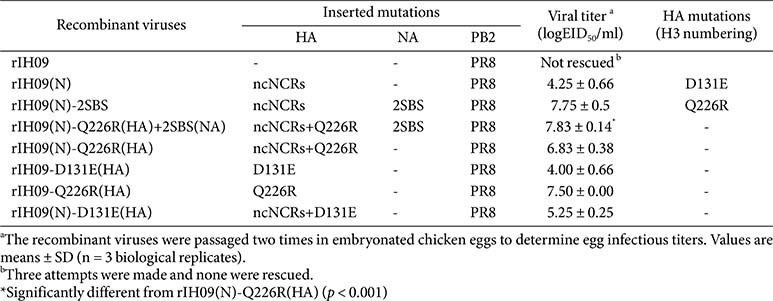

**Table 2:** Comparison of 2SBS of NA between avian H1N1 and early pdm09 strain.

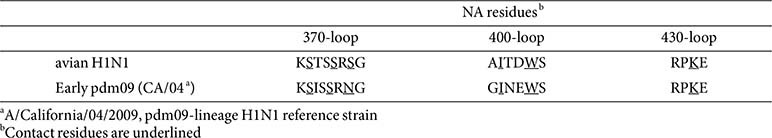

**Table 3:** Genomic characteristics and viral titers of recombinant IH09 viruses with avian-origin PB2.

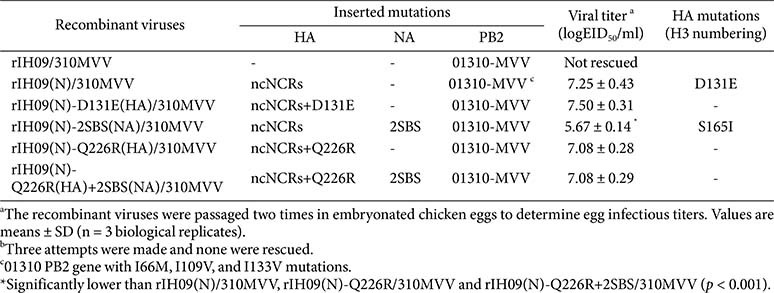

**Table 4:** Genomic characteristics and viral titers of recombinant IH09 viruses with cognate PB2.