# Supplementary material for: Genetic Optimization of Early 2009 Pandemic H1N1 Vaccine Strains for Improved Replication in Embryonated Chicken Eggs
Source: J Microbiol Biotechnol. 2025 Dec 15;35:e2510027. doi: 10.4014/jmb.2510.10027 (PMC12723478; doi:10.4014/jmb.2510.10027)
Supplement: Supplementary file 1 [file jmb-35-e2510027-supple.pdf]

J. Microbiol. Biotechnol. 2025. 35: e2510027

https://doi.org/10.4014/jmb.2510.10027

Supplementary Table and Figure

Table S1. Comparisons of major HA residues associated with receptor binding affinity and egg-adaptations.

**HA residue (H3 numbering)**	**Avian H1N1 strain[a]**	**Early strain (IH09, CA/09)[b]**	**Recent strain (GD19, Vic/22)[d]**	**Location in RBS[e]**	**Features**	**Ref**
**131**	E	D (E)[c]	D	130-loop	Increase receptor binding affinity (α2,3) (D131E)	[1-3]
**225**	G	D (G)[c]	D	220-loop	Increase receptor binding affinity (α2,3 and α2,6)<br>Increase viral titer in eggs (D225G)	[4-8]
**194**	L	L (I)[c]	L	190-helix	Increase receptor binding affinity (α2,3)<br>Increase viral titer in cells and eggs (L194I)	[9]
**212**	R	K (T)[c]	K	-	Increase viral titer in eggs (K212T)	[3]
**226**	Q	Q (R)[c]	Q	220-loop	Increase receptor binding affinity (α2,3)<br>Increase viral titer in eggs (Q226R)	[8, 10]

**133**	T	N	D	130-loop	Escape mutant<br>Increase receptor binding affinity (α2,3 and α2,6)	[11, 12]
**186**	P	S	P	-	Antigenic site<br>Increase receptor binding affinity (α2,3 and α2,6)	[2, 12, 13]
**188**	T	S	I	190-helix	Increase receptor binding affinity (α2,6)	[13]

[a] Consensus sequence of avian H1N1 isolates (total 711 complete HA protein sequences collected from GISAID database at 2025.02.14.).

[b] A/California/04/2009, pdm09-lineage H1N1 reference strain.

[c] Mutation acquired during egg passages.

[d] GD19, A/Guangdong-Maonan/SWL1536/2019 the recommended vaccine strain for 2020-2021; Vic/22, A/Victoria/4897/2022, the recommended vaccine strain for 2023-2025.

[e] 130-loop (134-138), 190-helix (188-200), 220-loop/helix (221-228).

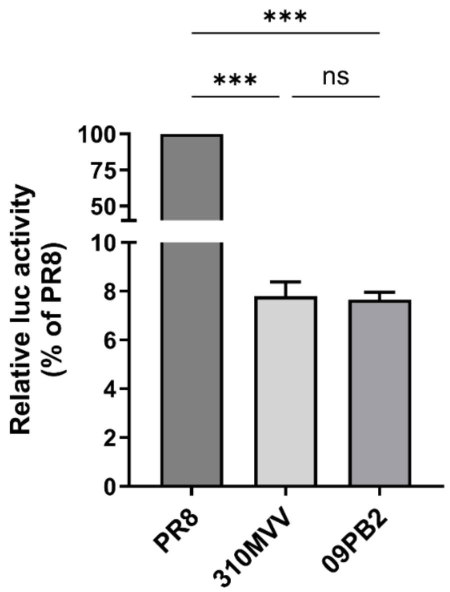

Fig. S1. Comparison of the effects of PR8 PB2, 310MVV PB2, and 09PB2 on the polymerase activities in 293T cells. The activities were compared using a mini-genome assay and the data were normalized as a percentage of polymerase activity of the PR8 PB2 gene. Each experiment was performed independently, and the data are presented as the mean ± SD of triplicate data from one experiment.

References

1. Sakabe S, Ozawa M, Takano R, Iwastuki-Horimoto K, Kawaoka Y. 2011. Mutations in PA, NP, and HA of a pandemic (H1N1) 2009 influenza virus contribute to its adaptation to mice. *Virus Res.* **158:** 124–129.
2. O'Donnell CD, Vogel L, Wright A, Das SR, Wrammert J, Li G-M*, et al.* 2012. Antibody Pressure by a Human Monoclonal Antibody Targeting the 2009 Pandemic H1N1 Virus Hemagglutinin Drives the Emergence of a Virus with Increased Virulence in Mice. *mBio* **3:** 10.1128/mbio.00120–00112.
3. Wang W, Lu J, Cotter CR, Wen K, Jin H, Chen Z. 2013. Identification of Critical Residues in the Hemagglutinin and Neuraminidase of Influenza Virus H1N1pdm for Vaccine Virus Replication in Embryonated Chicken Eggs. *J. Virol.* **87:** 4642–4649.
4. Takemae N, Ruttanapumma R, Parchariyanon S, Yoneyama S, Hayashi T, Hiramatsu H*, et al.* 2010. Alterations in receptor-binding properties of swine influenza viruses of the H1 subtype after isolation in embryonated chicken eggs. *J. Gen. Virol.* **91:** 938–948.
5. Liu Y, Childs RA, Matrosovich T, Wharton S, Palma AS, Chai W*, et al.* 2010. Altered Receptor Specificity and Cell Tropism of D222G Hemagglutinin Mutants Isolated from Fatal Cases of Pandemic A(H1N1) 2009 Influenza Virus. *J. Virol.* **84:** 12069–12074.
6. Chutinimitkul S, Herfst S, Steel J, Lowen AC, Ye J, Riel Dv*, et al.* 2010. Virulence-Associated Substitution D222G in the Hemagglutinin of 2009 Pandemic Influenza A(H1N1) Virus Affects Receptor Binding. *J. Virol.* **84:** 11802–11813.
7. Abed Y, Pizzorno A, Hamelin M-E, Leung A, Joubert P, Couture C*, et al.* 2011. The 2009 Pandemic H1N1 D222G Hemagglutinin Mutation Alters Receptor Specificity and Increases Virulence in Mice but Not in Ferrets. *J. Infect. Dis.* **204:** 1008–1016.
8. Carbone V, Schneider EK, Rockman S, Baker M, Huang JX, Ong C*, et al.* 2015. Molecular Characterisation of the Haemagglutinin Glycan-Binding Specificity of Egg-Adapted Vaccine Strains of the Pandemic 2009 H1N1 Swine Influenza A Virus. *Molecules* **20:** 10415–10434.
9. Yang L, Cheng Y, Zhao X, Wei H, Tan M, Li X*, et al.* 2019. Mutations associated with egg adaptation of influenza A(H1N1)pdm09 virus in laboratory based surveillance in China, 2009–2016. *Biosafety Health* **1:** 41–45.
10. Suptawiwat O, Jeamtua W, Boonarkart C, Kongchanagul A, Puthawathana P, Auewarakul P. 2013. Effects of the Q223R mutation in the hemagglutinin (HA) of egg-adapted pandemic 2009 (H1N1) influenza A virus on virus growth and binding of HA to human- and avian-type cell receptors. *Acta Virol.* **57:** 333–338.

11. Rudneva IA, Timofeeva TA, Mukasheva EA, Ignatieva AV, Shilov AA, Burtseva EI*, et al.* 2018. Pleiotropic effects of hemagglutinin amino acid substitutions of influenza A(H1N1)pdm09 virus escape mutants. *Virus Res.* **251:** 91–97.

12. Lee N, Khalenkov AM, Lugovtsev VY, Ireland DD, Samsonova AP, Bovin NV*, et al.* 2018. The use of plant lectins to regulate H1N1 influenza A virus receptor binding activity. *PLoS One* **13:** e0195525.

13. Vries RPd, Vries Ed, Martínez-Romero C, McBride R, Kuppeveld FJv, Rottier PJM*, et al.* 2013. Evolution of the Hemagglutinin Protein of the New Pandemic H1N1 Influenza Virus: Maintaining Optimal Receptor Binding by Compensatory Substitutions. *J. Virol.* **87:** 13868–13877.
